# Floral structure of two species of *Bulbophyllum* section *Cirrhopetalum* Lindl.: *B. weberi* Ames and *B. cumingii* (Lindl.) Rchb. f. (Bulbophyllinae Schltr., Orchidaceae)

**DOI:** 10.1007/s00709-016-1034-3

**Published:** 2016-10-31

**Authors:** Agnieszka K. Kowalkowska, Sławomir Turzyński, Małgorzata Kozieradzka-Kiszkurno, Natalia Wiśniewska

**Affiliations:** Department of Plant Cytology and Embryology, University of Gdańsk, Wita Stwosza 59, 80-308 Gdańsk, Poland

**Keywords:** *Bulbophyllum weberi*, *Bulbophyllum cumingii*, *Bulbophyllum*, *Cirrhopetalum*, Nectary, Osmophore, Ultrastructure

## Abstract

Flowers of *Bulbophyllum weberi* and *B. cumingii* are characterized by fly-pollinated features. The secretory activity was described in dorsal sepals in both species (putative osmophores), petals in *B. weberi* (possible osmophores) and adaxial surface of lips in both species. In the cells of dorsal sepals and petals of *B. weberi* proteins, dihydroxyphenols, lipids and starch grains were detected, in lateral sepals—lipids. Whereas in dorsal sepal of *B. cumingii* only lipids and starch grains were noted, in lateral sepals—proteins and dihydroxyphenols and in petals—proteins and starch grains. The lips in both species differed histochemically and ultrastructurally. The epidermal cells of lip groove in *B. weberi* contained lipids, proteins, starch grains in cytoplasm, dihydroxyphenols in vacuoles and pectic acids/mucilage on surface. Whereas in *B. cumingii*—few lipids, starch grains, no proteins, no dihydroxyphenols and no mucilage were noted. Ultrastructurally, in *B. weberi*, the secretory material was present on surface and vesicles building into plasmalemma, while in *B. cumingii*—cell wall ingrowths and microchannels in cuticle. The osmiophilic irregular materials and globular, osmiophilic globules in *B. weberi* are probably tannin-like materials. For the first time, we described the cell wall ingrowths in *Bulbophyllum* species: in lip of *B. cumingii* and petals of *B. weberi*.

## Introduction

*Bulbophyllum* Lindl. is a large, pantropical genus, containing about 2200 species, distributed from Continental Africa, Madagascar, East Indian Islands, Asia, Australia and the tropical Pacific islands to the Neotropics. Madagascar and New Guinea are the main centres of diversity (Pridgeon et al. 2014). Their flowers are dull cream or yellow-green to purple-brown, frequently spotted and hirsute, with mobile labella and appendages, emitted fruity or malodorous and carrion-like scents (Tan and Nishida 2000, 2005; Tan et al. 2002). The species demonstrate flowers, which are primarily pollinated by flies (van der Pijl and Dodson 1966; van der Cingel 2001; Nishida et al. 2004): Diptera: Calliphoridae, Sarcophagidae, Platystomatidae and Milichiidae, species from genus *Bactrocera* from Tephritidae and *Drosophila* from Drosophilidae), but also by bees, wasps and foraging beetles (Stpiczyńska et al. 2015 and references therein).

Fly pollination is understood to comprise two syndromes: myophily and sapromyophily. The set of features characterizing myophilous flowers includes simple, relatively small flowers with bright dull colour (yellow-green to brownish or purplish) and musky, slightly sweet or fruity odour. It is also known that myophilous flowers could produce secretion in open shallow nectaries which occur in longitudinal labellar groove in *Bulbophyllum* family (Endress 1994; Kite et al. 1998; Jürgens et al. 2006; Johnson and Jürgens 2010; Humeau et al. 2011; Davies and Stpiczyńska 2014; Stpiczyńska et al. 2015). Whereas the sapromyiophilous pollination syndrome embraces dark flowers (brown, purple, greenish, often with great depth), emitting the putrescent odour containing sulphur compounds (Feinstein et al. 2009; van der Pijl and Dodson 1966). In fruit fly-pollinated *Bulbophyllum* species, methyl eugenol (aromatic compound) and ketones have been identified as key chemical components in plant-pollinator interaction. In *B. cheiri* methyl eugenol acts as sex pheromone precursor for males of *Bactrocera* species during pollination (Tan et al. 2002; Nishida et al. 2004). The flowers of Malaysian *Bulbophyllum vinaceum* also emit methyl eugenol and attract *Bactrocera dorsalis* and *B. unimacula* (Tan et al. 2006). Furthermore, raspberry ketone was detected as the major fragrance compound in *Bulbophyllum apertum* ssp. *verrucosum* being attracted to *Bactrocera* species sensitive to this ketone (Tan and Ritsuo 2005). Zingerone, identified in *Bulbophyllum baileyi* and *B. patens*, was also qualified as volatile component enticing *Bactrocera* species (Tan and Nishida 2000, 2007). The unpleasant urine-like odour in sapromyiophilous *Bulbophyllum variegatum* could be ascribed to indole, *p*-cresol, 2-heptanone (Arctander 1994; Dobson 2006) and nitrogenous and sulphur compounds (Humeau et al. 2011).

The labellar morphology of *Bulbophyllum* species has homogenous and highly conservative characters (Teixeira et al. 2004): the linguiform mid-lobe with adaxial median longitudinal groove, relatively small and forwardly pointing lateral lobes and also the articulation to the column foot allowed lip to balance. However, the micromorphology and anatomy of species from section *Didactyle* showed considerable variation (Nunes et al. 2014). The anatomical studies in up-to-date investigated species revealed the general lip arrangement from the transverse sections: the single layer of epidermis, few layers of subepidermis and ground parenchyma built by the isodiametric cells with collateral vascular bundles. The labellar ground parenchyma was fleshy without intracellular spaces, e.g. *Bulbophyllum exaltatum* Lindl., *B. involutum* Borba, Semir and F. Barros, *B. meridense* Rchb.f., *B. perii* Schltr., *B. popayanense* Kraenzl., *B. tripetalum* Lindl., *B. weddellii* (Lindl.) Rchb.f. (Nunes et al. 2014), *B. careyanum* (Hook.) Spreng., *B. morphologorum* Kraenzl., *B. orientale* Seidenf., *B. wangkaense* Seidenf. (Davies and Stpiczyńska 2014) or with intracellular spaces, e.g. *B. wendlandianum* (Kraenzl.) Dammer (Kowalkowska et al. 2015), *B. falcatum* (Lindl.) Rchb.f., *B. lupulinum* Lindl., *Bulbophyllum maximum* (Lindl.) Rchb.f., *B. schinzianum* Kraenzl. (Stpiczyńska et al. 2015). The idioblasts with raphides occurred under the epidermal cells through the subepidermis and parenchyma (Davies and Stpiczyńska 2014; Kowalkowska et al. 2015). In petals, lip epichile, gynostemium and ovary of certain Neotropical bulbophyllums, the idioblasts with raphides and cellulosic helical wall thickenings were visible (Nunes et al. 2014).

The epidermis of the adaxial labellar groove contained isodiametric cells, e.g. in *B. meridense* (Nunes et al. 2014), *B. wendlandianum* (Kowalkowska et al. 2015) or palisade-like cells, e.g. in *B. involutum* (Nunes et al. 2014), *B. careyanum*, *B. morphologorum*, *B. orientale* and *B. wangkaense* (Davies and Stpiczyńska 2014). It is known that the secretion in *Bulbophyllum* flowers is produced superficially on the lip (van der Pijl and Dodson 1966; Teixeira et al. 2004; Kowalkowska 2009; Davies and Stpiczyńska 2014; Nunes et al. 2014; Kowalkowska et al. 2015; Stpiczyńska et al. 2015). In most representatives, this secretion was described as nectar, but there is evidence that in some species, the secretion could be oil in conjunction with sugar (Pohl 1935; van der Cingel 2001; Nunes et al. 2014) or protein-rich mucilage (Davies and Stpiczyńska 2014). The oily or resinous material has been described in the Asian species of *Bulbophyllum*, i.e. in *B. lobbii* Lindl. (section *Sestochilus* (Breda) Benth. and Hook.f.), *B. macranthum* Lindl. (section *Stenochilus* J.J.Sm.), *B. alticola* Schltr. (section *Codonosiphon* Schltr.), *B. auratum* (Lindl.) Rchb.f. (section *Recurvae*). Pohl (1935) recorded that the flower of *B. lobbii* and *B. macranthum* produces oil and sugar at the base of lip, in the latter also by adjacent floral parts. The glistening droplets of sticky fluid on lips a few hours after opening have been found in *B. alticola* from Papua New Guinea (Jongejan 1994). Flowers of *B. auratum* produce a huge amount of sticky fluid, which functions as a trap for insects. The smaller ones draw down, whereas the bigger ones, after trying to release, are thrown against the column by hinged lip regaining to its original position (Jongejan 1994).

The lip of *Bulbophyllum* species contains adaxial secretory tissue, which is defined in the literature as ‘nectary’. Teixeira et al. (2004) and Nunes et al. (2014) described both nectaries in lip groove and osmophores on the lip lobes of Neotropical species of *Bulbophyllum*. The liquid-like nectar was present in the lip cavity (=groove) of the non-wind-assisted fly-pollinated *Bulbophyllum* species: *B. epiphytum* (Barb. Rodr.) Cogn., *B. glutinosum* (Barb. Rodr.) Cogn., *B. regnellii* Rchb.f. and *B. rothschildianum* (O’Brien) J.J. Sm., whereas the osmophores were located as papillae on the lip lobes and in the upper surface of the lip callus in most investigated wind and non-wind-assisted species (Teixeira et al. 2004). The labellum in *B.* section *Didactyle* (Nunes et al. 2014) was clothed with trichomes (possible osmophores), with a secretory cavity in the callus (nectary), bound by scale-like papillae. With some differences between species, the epidermal papillose cells on hypochile gave positive reaction on lipids and formed the boundary of secretory cavity. The three to five subepidermal secretory layers with cytoplasmic proteins were described in callus cavity. The co-occurrence of osmophores and nectaries was in highest probability related to the pollination mechanism of those *Bulbophyllum* species. Flies are enticed to flowers by volatile oils exuded on lip surface (Da Silva et al. 1999) and walking on the lip could feed on nectar secreted in the lip cavity (Borba and Semir 1998). Previously, Vogel (1990) noted two heterogenous fragrance centres in *B. ornatissimum* (Rchb. f.) J.J. Sm.: the movable lip with a trimethylamine-like odour and the upper groove with a viscous nectar. The fragrances generally are not accumulated on the surface (Vogel 1990) or the meagre amount was noticed (Stern et al. 1987; Vogel 1990; Kowalkowska et al. 2012). The labellar secretion consisted of protein in a matrix of mucilage (without lipids) was described in four species of section *Racemosae* (Davies and Stpiczyńska 2014). It was hypothesised that mucilage precursors are produced by the dictyosomes of subepidermal cells and may pass along the symplast via plasmodesmata into the adjoining palisade-like cells. The latter contained abundant profiles of rough endoplasmic reticulum (RER), in lack of smooth endoplasmic reticulum (SER), which indicated for protein synthesis in secretory cells rather than for lipid synthesis. The presence of dictyosomes in epidermis and subepidermis corroborated the above hypothesis, as the dictyosomes are involved in carbohydrate metabolism, particularly in mucilage production. The mucilage, after chemical modification, forms a mucilaginous matrix, which facilitates transport for protein produced by RER. Then, such material is transported into vesicles through cytoplasm, transverses the cell wall, and accumulates between the cell wall and cuticle-forming blisters (described also as dilatations, distensions, swellings). Such blisters may rupture releasing material onto the cuticle surface outside the cells. The other routes of releasing the secretory material are through cuticular pores/cracks/tears or microchannels in cuticle (in the floral osmophores fragrance production of *Passiflora suberosa* L. (García et al. 2007), *Anacamptis pyramidalis* f. *fumeauxiana* (Kowalkowska et al. 2012), *Chloraea membranacea* Lindl. (Sanguinetti et al. 2012), *Bulbophyllum wendlandianum* (Kraenzl.) Dammer (Kowalkowska et al. 2015).

The representatives from section *Cirrhopetalum* Lindl. (according to Pridgeon et al. 2014) are characterized by a many-flowered inflorescence with flowers arranged in subumbellate raceme. The dorsal sepal is free, 3–5-veined, with entire or distally erose margins. The 3–5-veined lateral sepals are twice to 5.5 times as long as the dorsal, twisted near the base so that the upper margins turn inward, connate along their upper margins. Petals are 3-veined with fimbriate margins. The lip is mobile on a thin ligament, undivided or auriculate. *Bulbophyllum weberi* grows as epiphyte or lithophyte in the Philippines between 100 and 1500 m a.s.l. *B. cumingii* is epiphyte growing from 50 to 500 m a.s.l. in the Philippines and Malaysia. In this study, we would like to describe secretory activity in flowers, based on micromorphological and anatomical studies (histochemistry, ultrastructure) as well as to discuss the results. The reasons to compare the results of both species are as follows: (1) *B. weberi* and *B. cumingii* are plants from the same section growing in the same range, (2) the same colours of flowers: yellow and purple, but in different arrangement, (3) coverage by papillae of dorsal sepals and petals, with some differences and (4) morphological similarities in lips shapes and features. The above-mentioned macromorphological features let us ask the question whether they are the same on anatomical level.

## Materials and methods

**Fig. 1:**
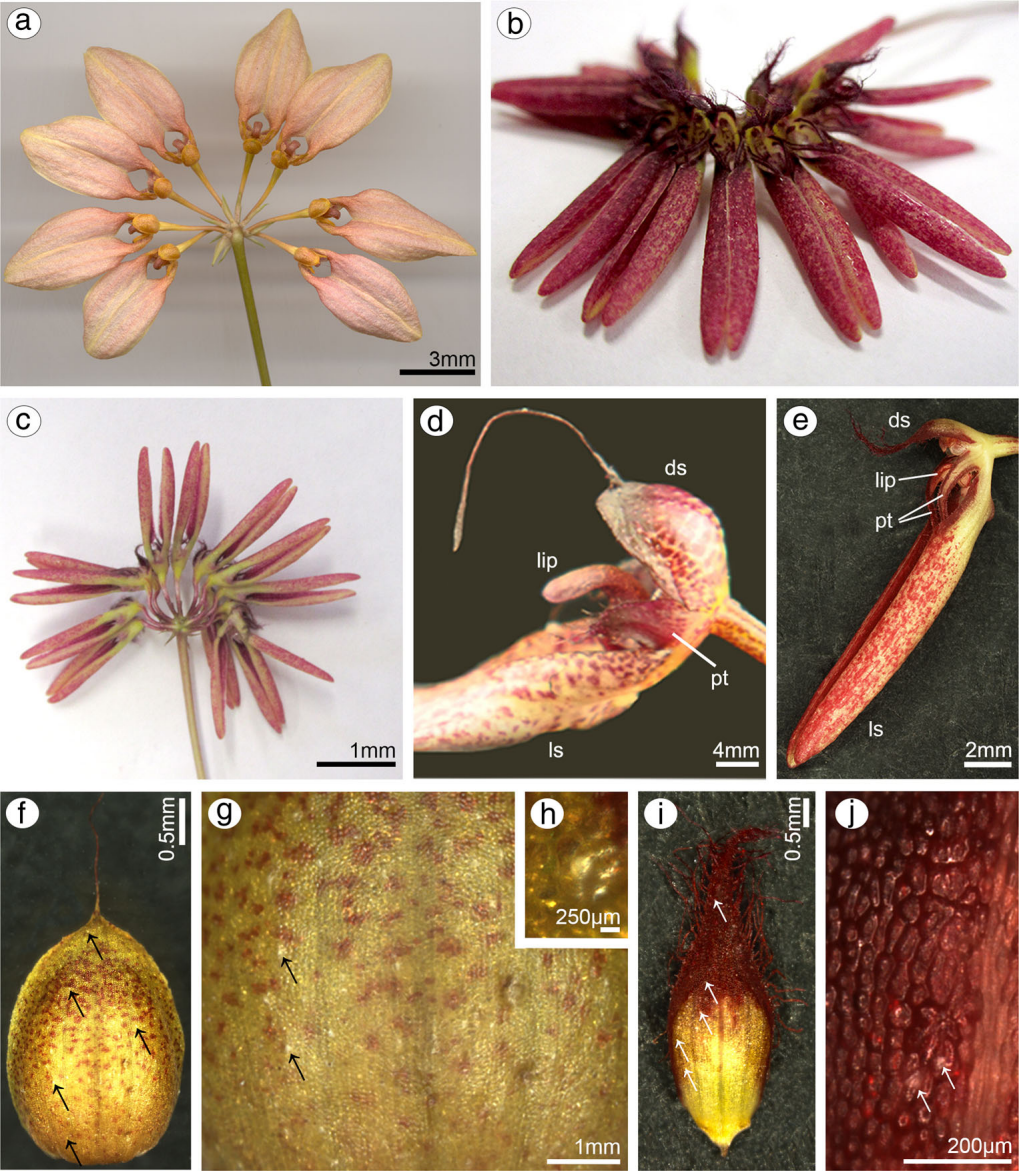
Macromorphological features of flowers of *Bulbophyllum weberi* and *B. cumingii.* **a** Inflorescence of *B. weberi* (phot. by A. Sieder); **b** inflorescence of *B. cumingii* (front view); **c** inflorescence of *B. cumingii* with lateral sepals free at the base (bottom view); **d** single flower of *B. weberi* and **e** *B. cumingii:* dorsal sepal (*ds*), lateral sepal (*ls*), petal (*pt*), lip; **f** dorsal sepal of *B. weberi* with idioblasts with raphides emerged from the tissue (*arrows*); **g**, **h** detail of dorsal sepal of *B.* weberi; **i** dorsal sepal of *B. cumingii*, *arrows* showing idioblasts with raphides; **j** detail of dorsal sepal of *B. cumingii*

Samples were collected from flowers at anthesis: *B. weberi* in November 2011 (Fig. 1a; voucher number ORCH 92324 and *B. cumingii* in April 2012 (Fig. 1b, c; voucher number 098B 227-1) in Botanischer Garten der Universität Wien (WU). The identification of flowers was done based on: Seidenfaden (1973, 1979), database of Swiss Orchid Foundation (Herbarium Jany Renz), *B. cumingii*, specimen seen: K, spirit collection 49431.000 (the Herbarium of the Royal Botanic Gardens, Kew, UK). Fresh flowers were observed under a Nikon SMZ1500 stereomicroscope (light microscope (LM)). Plant material was fixed in 2.5 % glutaraldehyde (GA) in 0.05 M cacodylate buffer (pH = 7.0). The material for LM was rinsed with cacodylate buffer and then dehydrated in acetone (for TEM) or ethanol series (for LM). Whole dehydrated material was embedded in epoxy resin (Spurr 1969) and methylmethacrylate-based resin (Technovit 7100, Heraeus Kulzer GmbH). Sections were cut with glass knives (1–5-μm thick) and mounted on glass slides. For LM, the material was stained with 0.05 % Toluidine Blue O (TBO) for 1 min at 60 °C on a hot plate (Feder and O’Brien 1968; Ruzin 1999). Aniline Blue Black (ABB, C.I. 20470) was used for detection of water-insoluble proteins (Jensen 1962). The periodic acid-Schiff reaction (PAS reaction) was used to identify the presence of water-insoluble polysaccharides (Jensen 1962) and Sudan Black B (SBB) for lipid localization (Bronner 1975). The preparations were examined and photographed with a Nikon Eclipse E 800 light microscope and a Nikon DS-5Mc camera using the Lucia Image software. A 0.05 % (*w*/*v*) aqueous ruthenium red solution and a 10 % (*w*/*v*) aqueous solution of FeCl_3_ were used to test for pectic acids/mucilage (Johansen 1940) and catechol-type dihydroxyphenols (Gahan 1984), respectively. The sections from these stainings were observed using the differential interference contrast (DIC) imaging.

For scanning electron microscopy (SEM), after dehydration in an ethanol series, the samples were dried by the critical point method using liquid CO_2_, coated with gold and observed in a Philips XL-30.

For transmission electron microscopy (TEM), the floral material was fixed in glutaraldehyde (2.5 % GA) in 0.05 M cacodylate buffer (pH 7.0). The material was post-fixed overnight in 1 % OsO4 in cacodylate buffer in a refrigerator and then rinsed in the buffer. After 1 h in 1 % solution of uranyl acetate in distilled water, the material was dehydrated with acetone and embedded in Spurr’s resin. Ultrathin sections were cut on a Sorvall MT 2B ultramicrotome with a diamond knife and contrast stained with uranyl acetate and lead citrate. The sections were examined in a Philips CM 100 transmission electron microscope. Samples were prepared in accordance with procedures described elsewhere (Kowalkowska et al. 2012, 2014).

## Results

### Dorsal sepal

**Fig. 2:**
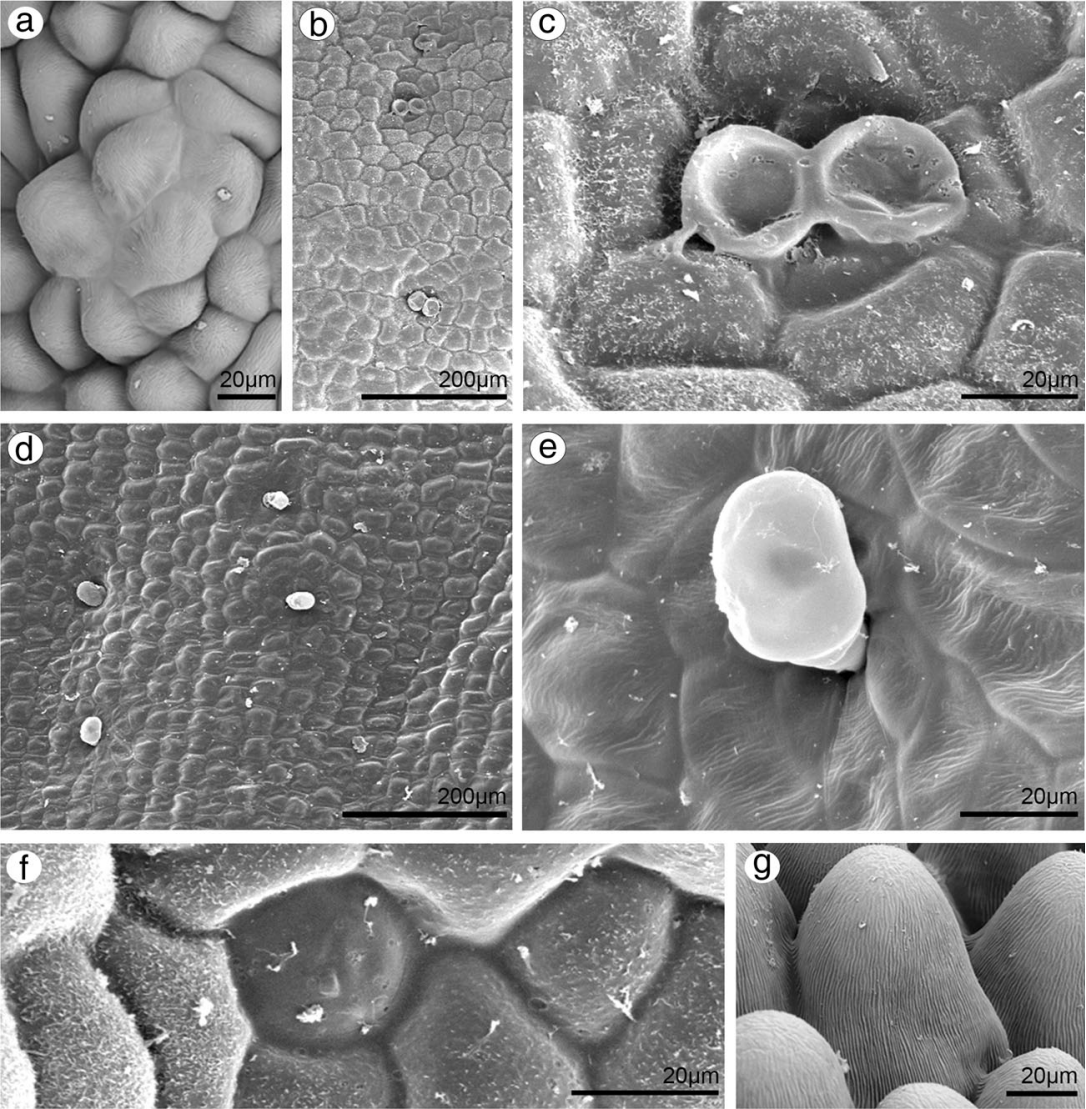
Micromorphological features of dorsal sepal of *B. weberi* (SEM). **a** Elevated groups of cells; **b** the adaxial (outer) surface of dorsal sepal with residues of secretory material and sunken unicellular trichomes (**c**); **d** the inner surface of dorsal sepal with bicellular trichomes (**e**); **f** stomata present on the outer surface; **g** conical papillae with striate wall pattern on apical part of the sepal

**Fig. 3:**
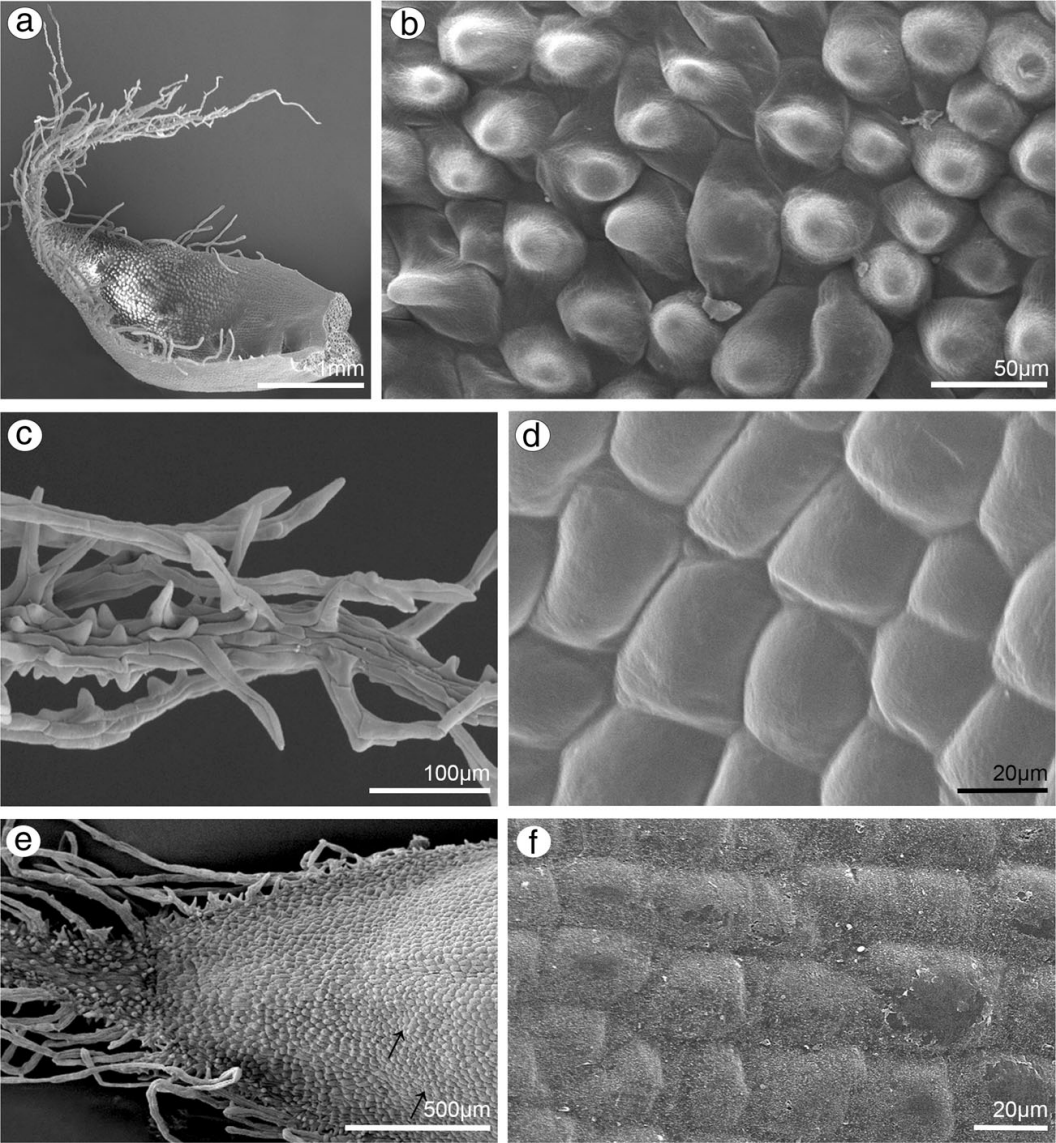
Micromorphological features of dorsal sepal of *B. cumingii* (SEM). **a** Adaxial (inner) side of dorsal sepal with conical papillae at apex (**b**), multicellular trichomes on the margins (**c**) and smooth cells at base (**d**); **e** abaxial (outer) side of dorsal sepal with trichomes on margins, conical papillae at apex, groups of elevated cells (*arrows*) and smooth cells with residues of secretions at base (**f**)

**Fig. 4:**
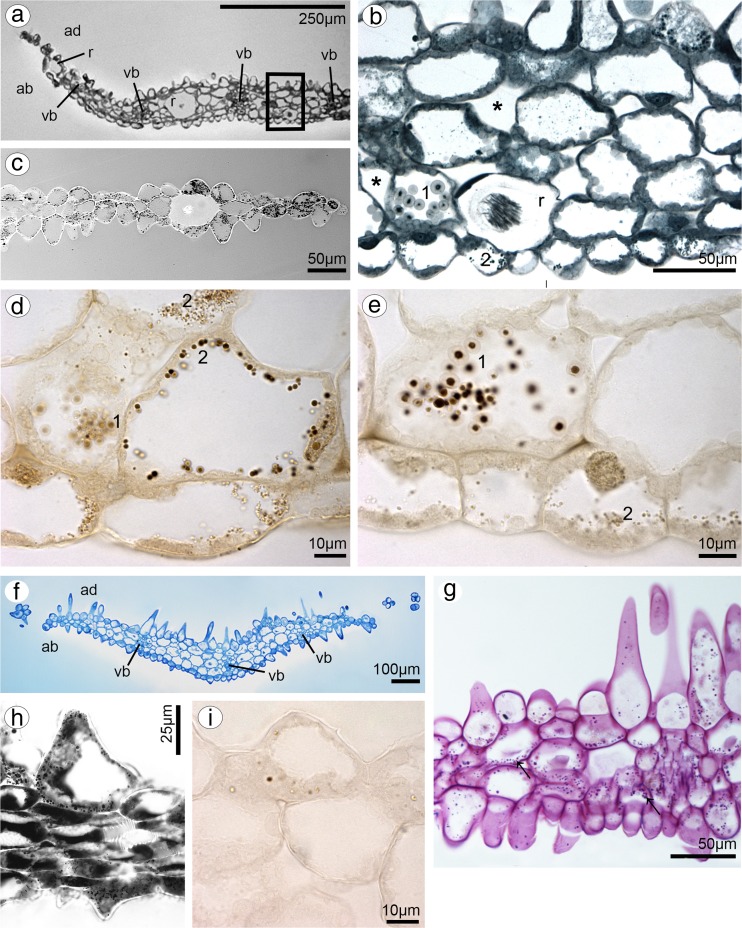
Anatomical features of transverse section of dorsal sepals (LM). **a** *B. weberi*: a single layer of epidermis and parenchyma with vascular bundles (*vb*), idioblasts with raphides (*r*) (*ad* adaxial side, *ab* abaxial side, TBO); **b** detail of *B. weberi* (**a**), presenting two types of globules: bigger ones (*1*) and smaller ones (*2*), *asterisks* intercellular spaces, *r* raphides (ABB); **c** lipid bodies after SBB treatment; **d, e** two types (*1*, *2*) of globules stained selectively on dihydroxyphenols (FeCl_3_); **f** *B. cumingii*: a single layer of epidermis with longer papillae, than in *B. weberi*, with vascular bundles (*vb*) (TBO); **g** numerous tiny starch grains (*arrows*, PAS); **h** lipid bodies stained with SBB; **i** no globules staining with FeCl_3_

**Fig. 5:**
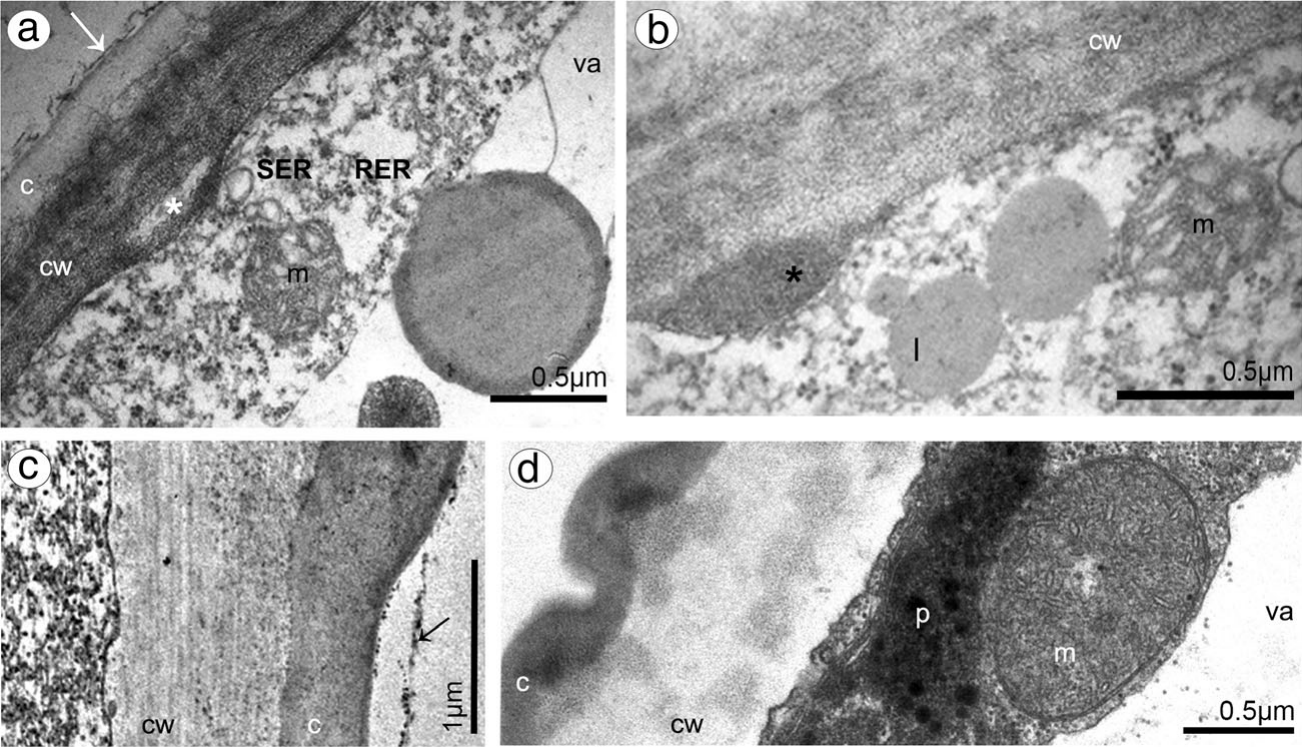
Ultrastructural features of dorsal sepals (TEM). **a**, **b** In *B. weberi*: the cytoplasm containing mitochondria (m), SER, RER, lipid bodies (l), free ribosomes, the vacuole (va) with osmiophilic globules, material protruding into the cell interior, sometimes electron-dense (*asterisk*) and the meagre amount of exudates (*white arrow*) on the cuticle surface (*c*), *cw* cell wall; **c**, **d** in *B. cumingii*: the cytoplasm with well-organized mitochondrion (*m*), plastid (*p*) with plastoglobuli and irregular profiles (probably chromoplasts), free ribosomes, sometimes indicated free residues (*arrow*) on cuticle (*c*), *cw* cell wall and *va* vacuole

Dorsal sepal of *B. weberi* (Fig. 1d, f–h) was entirely yellow with purplish blotches, also on long multicellular hair at apical part. In *B. cumingii* (Fig. 1e, i–j), dorsal sepal was also yellow, but with red/purple apical part and numerous red/purple hairs. In both species, the conspicuous idioblasts with raphides emerged from the tissue on the whole outer dorsal sepal surface (Fig. 1f–j), visible in SEM as elevated groups of cells (Figs. 2a, 3e). Such elevations were caused by idioblasts growth and increasing of the cell size. In *B. weberi*, the both surfaces of dorsal sepal (Fig. 2b–e) were smooth, with a few minute sunken unicellular (situated in pairs) or bicellular trichomes in small depressions. The apical part of the sepal abaxial (outer) surface bore stomata, placed also in the depressions (Fig. 2f). The residues of secretory material were detected on the outer (abaxial) slightly rugose surface, also on trichomes and stomata (Fig. 2c, f). Close to the hair, flat cells were transformed into conical papillae with striate wall pattern (Fig. 2g), also on apical hair. In *B. cumingii*, both surfaces were also smooth (Fig. 3d, f), besides the apical part with conical papillae with striate wall pattern (Fig. 3a, b, e) and on margins with multicellular trichomes (Fig. 3a–e). In this species, sunken trichomes and stomata in depressions were not occurred. SEM images showed the residues of secretions on the cells of outer surface, the same as in former species (Fig. 3f). On the transverse section, the tissue consisted of a single layer of epidermis and parenchyma built by irregular shaped cells with small intercellular spaces (small in *B. weberi*, larger in *B. cumingii*) and several vascular bundles (Fig. 4a, b, f). In *B. cumingii*, the inner (adaxial) surface was consisted of longer conical papillae (Fig. 4f). The dorsal sepal apical hair and tissue, in the first species had two types of globules (Fig. 4d, e). The first (bigger ones: with darker centre and lighter edge) and second (smaller) ones were stained selectively on proteins (Fig. 4b) and dihydroxyphenols (Fig. 4d, e). The first ones seemed to be plastids with electron-dense body (compare with results from TEM for petals Fig. 9g). The second ones appeared in TEM studies (Fig. 5a) as the vacuolar osmiophilic globules. Testing with PAS and SBB, tiny starch grains (Fig. 4g) and lipid bodies (Fig. 4c, h) revealed in the dorsal sepal tissue in both species, respectively. Despite of similarities between two species, the histochemical stainings on proteins (ABB) and dihydroxyphenols (FeCl_3_, Fig. 4i) in *B. cumingii* did not indicate the presence of any globules. Ultrastructurally, the cytoplasm of *B. weberi* contained lipid bodies, well-organized mitochondria, smooth endoplasmic reticulum and free ribosomes (Fig. 5a, b). The cell wall comprised the material, sometimes electron-dense, protruding into the cell interior. The cuticle surface was covered by the meagre amount of exudates (Fig. 5a). In *B. cumingii*, the cytoplasm contained well-organized mitochondria, plastids with plastoglobuli and irregular profiles (probably chromoplasts) and free ribosomes (Fig. 5c, d). The presence of few residues on the apex of dorsal sepal, without cuticular channels or pores, was sometimes indicated (Fig. 5c).

### Lateral sepals

**Fig. 6:**
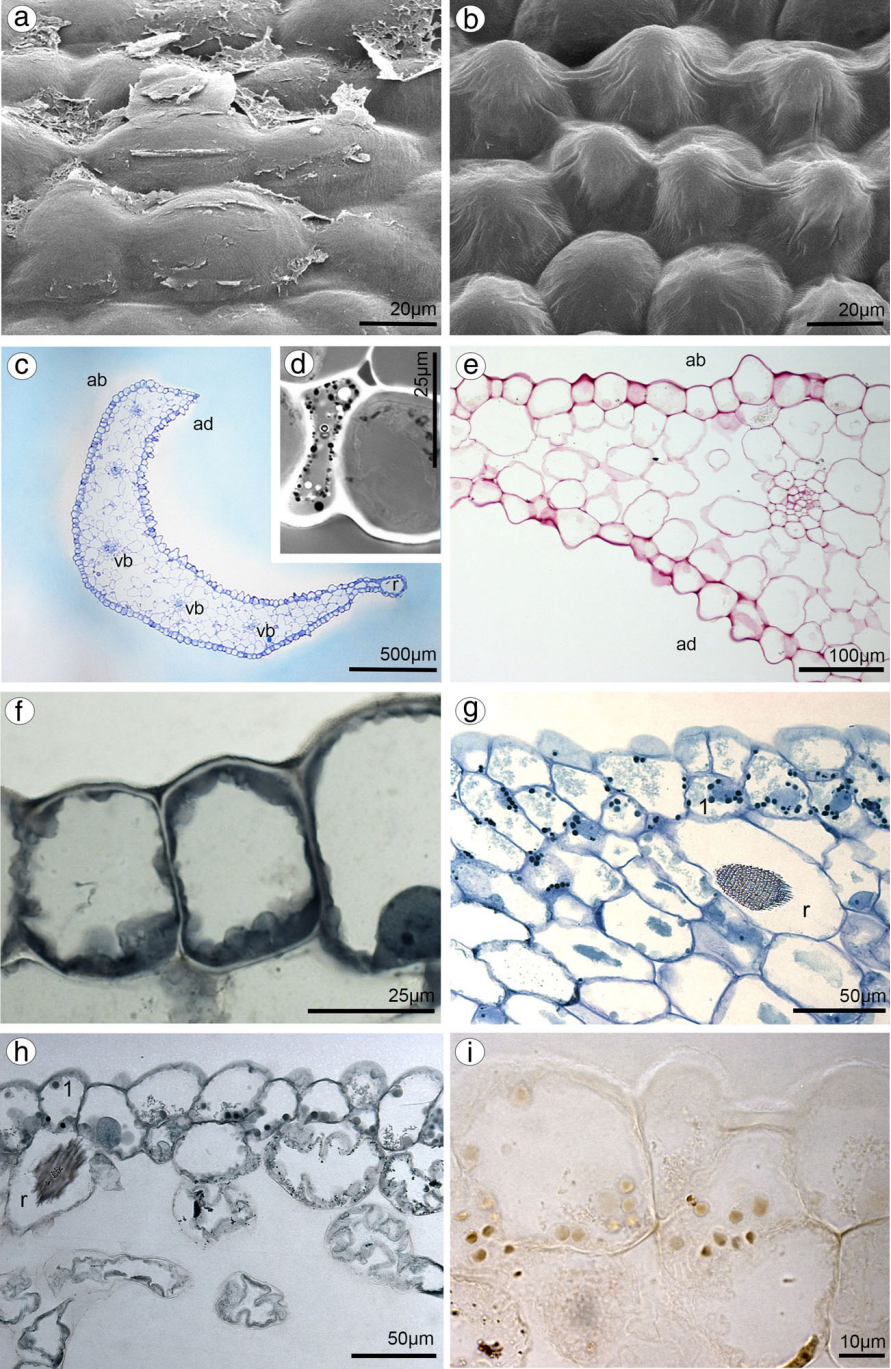
Micromorphological and anatomical features of lateral sepals. *B. cumingii*: **a** apex built of smooth cells with residues of secreted material (SEM); **b** basal part built by conical papillae with striate wall pattern (SEM); **c** transverse section of lateral sepal with few vascular bundles (*vb*), *r* idioblast with raphides, *ad* adaxial side, *ab* abaxial side (TBO); **d** few lipid droplets (SBB); **e** transverse section after treatment with ruthenium red, *ad* adaxial side, *ab* abaxial side; and after treatment with ABB (**f**); *B. weberi*: **g** idioblasts with raphides (*r*) and globules (*1*, TBO), visible after testing with ABB (**h**) and FeCl_3_ (**i**)

Lateral sepals were free at the base in both species (Fig. 1a, c). Both surfaces were built of smooth cells in two examined species, wherein the residues of secreted material covered the lateral sepals apices in *B. cumingii* (Fig. 6a). The sepals base was built by conical papillae with striate wall pattern (Fig. 6b, c). Staining with ruthenium red did not indicate the presence of mucilage on lateral sepals in both species (Fig. 6e), stained only cell walls. As in dorsal sepal, the idioblasts with raphides occurred in the first subepidermal layer of parenchyma in large quantities (Fig. 6g, h) in both species. Noticeably, the idioblasts were more numerous at the base and at apical parts of lateral sepals in *B. cumingii*. The cytoplasm comprised few tiny starch grains (not shown). Testing with SBB, lipoid layer on cells (cuticle) and few lipid droplets in cytoplasm were recorded (Fig. 6d). Treatment with ABB revealed globules stained on proteins in *B. weberi* (Fig. 6h, visible in control staining TBO—Fig. 6g), not in *B. cumingii* (Fig. 6f), the same as described in dorsal sepals. Some globules were also stained after treatment with FeCl_3_ (Fig. 6i).

### Petals

**Fig. 7:**
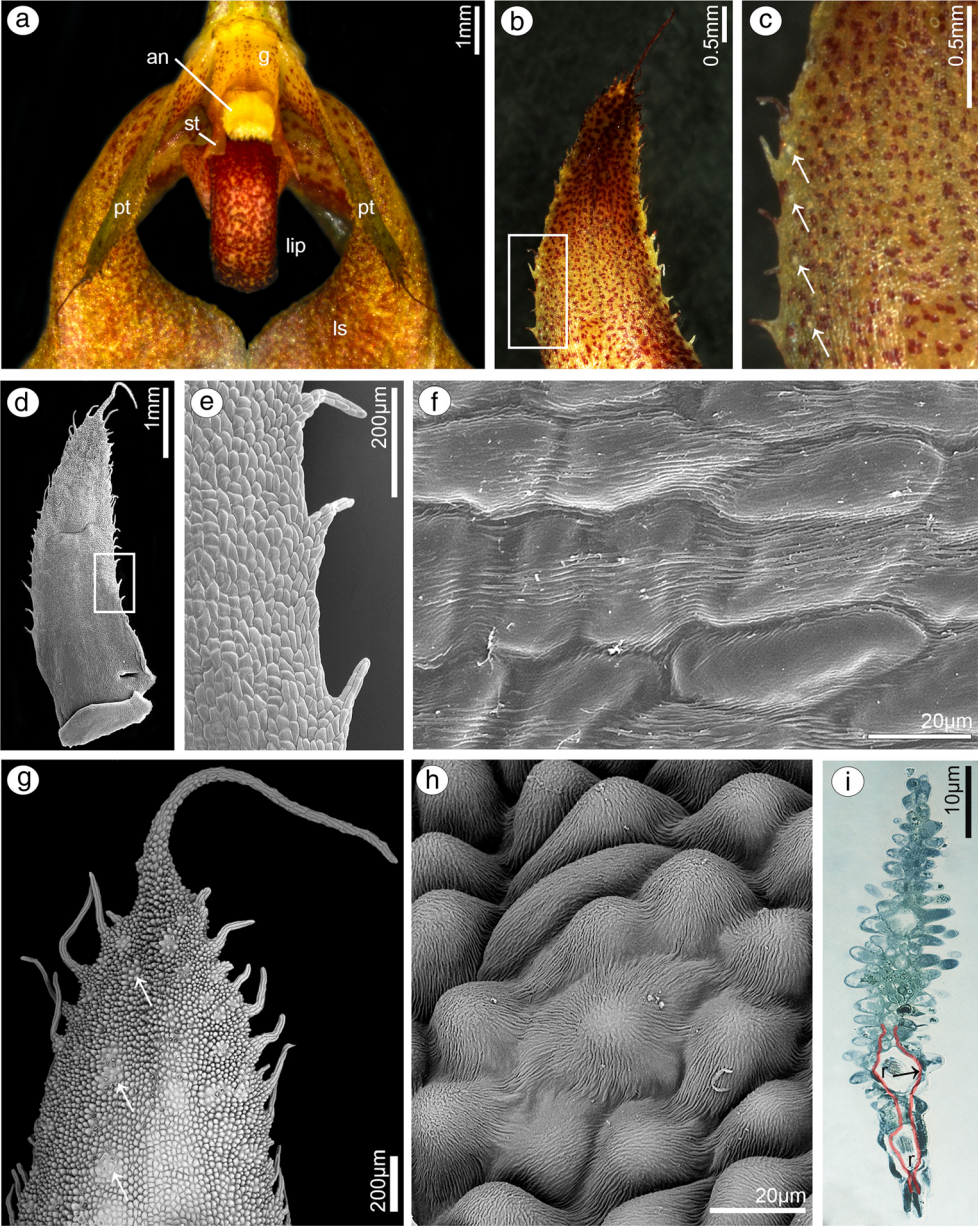
Floral features of petals of *B. weberi.* **a** Flower: *an* anther, *g* gynostemium, lip, *ls* lateral sepal, *pt* petal, *st* staminodium (LM); **b** yellow with purple blotches petal (LM); **c** detail of yellow with purple blotches petal (**b**) with idioblasts with raphides *(arrows)* (LM); **d** petal with single multicellular hair at apex and shorter ones at margins (**e**), smooth cells at the base (**f**), conical papillae with rounded tips towards the apex (**g, h**), with striate wall pattern (SEM); **g** the apex with large number of the elevated groups of cells (**h**) (SEM); **i** teitransverse section of apex with idioblasts containing raphides (*r*) increasing cell size (*marked with red line*)

**Fig. 8:**
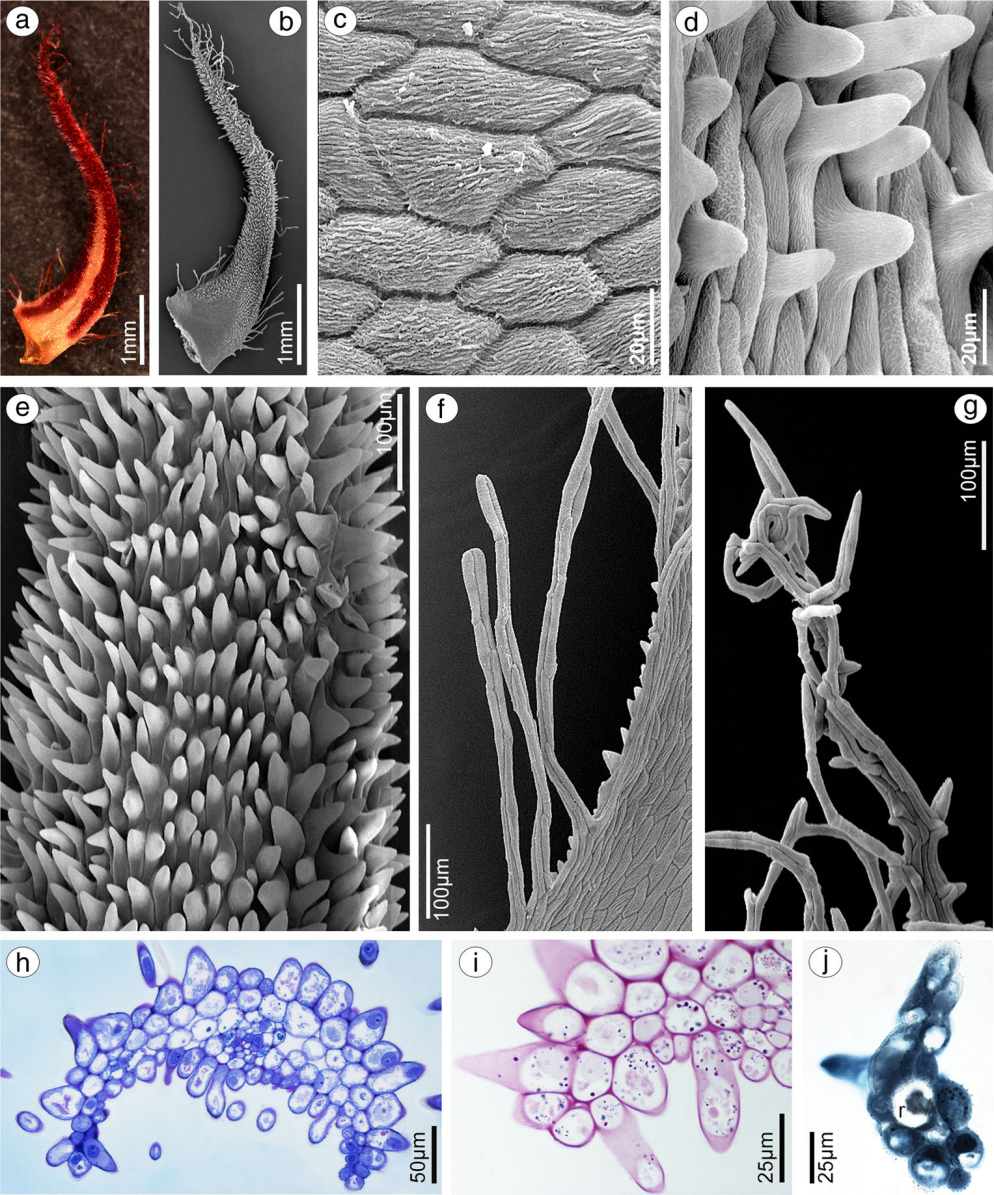
Floral features of petals of *B. cumingii.* **a** Petal yellow with red/purple margins (LM); **b** petal with smooth cells at base (**c**) and conical elongated papillae towards apex (**d, e**), with striate wall pattern, and few multicellular hairs at margins (**f**) and apex (**g**) (SEM); **h** transverse section of petal (TBO); **i** tiny starch grains (*arrows*, PAS); **j** the apex after treatment with ABB, idioblast with raphides (*r*)

**Fig. 9:**
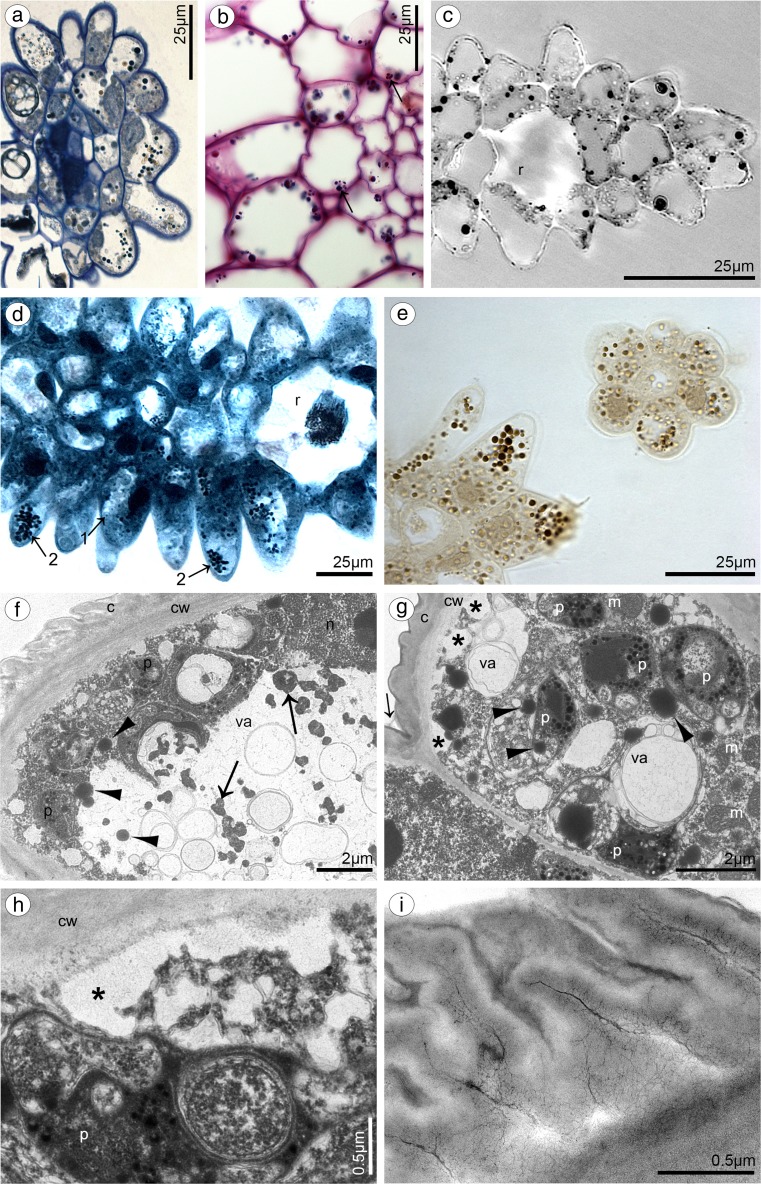
Histochemical and ultrastructural features of petals of *B. weberi* **a** transverse section of multicellular hair with globules (TBO); **b** numerous starch grains (PAS); **c** lipid bodies, raphides (*r*) (SBB); **d** two types of globules (*1*, *2*) after treatment with ABB; **e** globules stained on dihydroxyphenols (FeCl_3_); **f–h** ultrastructure of petal papillae (TEM): the ingrowths (*asterisks*) arising from the inner surface of the outer walls (*cw* cell wall), no (**f, i**) or a meagre amount of substances (**g**) on the cuticle (*c*) surface, large number of mitochondria (*m*), plastids (*p*) with plastoglobuli and electron-dense body, free ribosomes, vacuoles (*va*) with osmiophilic material (*arrows*) and globules (*arrowheads*, visible also in cytoplasm) (*n* nucleus); **i** microchannels visible in reticulate cuticle (TEM)

Comparing results from both species, few differences were noticeable. In *B. weberi*, petals were yellow with purple blotches (Fig. 7a–c), whereas in *B. cumingii*, also yellow, but with red/purple margins (Fig. 8a). The presence of single multicellular hair at the apex and shorter ones at margins was remarked in *B. weberi* (Fig. 7a, b, d, g), while in *B. cumingii*, the apex was prolonged with few multicellular hairs, which were also present on margins (Fig. 8a, b, g). In both species, the epidermal cells were smooth at the base (Figs. 7d–f; 8b, c) and towards the apex papillae predominated (Figs. 7d, g–i; 8d–e, h, i). Regardless the cell shape, the striate ornamentation was noticed on the whole surface (Figs. 7f, h; 8c, d). The other difference between species concerned the papillae density, their degree of coverage and shape. The conical papillae with rounded tips occurred in *B. weberi* (Fig. 7h), whereas in *B. cumingii* (Fig. 8d, e), they were conical and elongated. Equally with dorsal sepal of *B. weberi*, also in petals, the elevated groups of epidermal cells were seen in large number (Fig. 7g, h) and were caused by increasing size of raphides in idioblasts (Fig. 7i). In *B. cumingii* idioblasts with raphides were also noticed (Fig. 8j). The histochemical stainings revealed numerous tiny starch grains (Figs. 8i, 9b) and lipid bodies in *B. weberi* (Fig. 9c), not noted in *B. cumingii* (not shown). Some globules were stained on proteins (in *B. cumingii* Fig. 8j, in *B. weberi* Fig. 9a, d: the first and second types) and some on dihydroxyphenols (strongly in *B. weberi* Fig. 9e, slightly in *B. cumingii*). The TEM results of *B. weberi* showed that the striate cuticle was reticulate (with microchannels) (Fig. 9i); however, no (Fig. 9f) or a meagre amount of substances (Fig. 9g) appeared on the surface. In *B. cumingii* no secretory activity on surface was detected; though in SEM, a little residue of secretory material was perceived (Fig. 8c). In the former species, the cytoplasm revealed features of secretory cells such as large number of mitochondria, plastids with plastoglobuli, and electron-dense body, free ribosomes, osmiophilic irregular material in vacuoles and globular osmiophilic bodies in vacuoles, visible also in cytoplasm (Fig. 9f, g). The most remarkable feature of these cells was the observation of the ingrowths arising from the inner surface of the outer walls (Fig. 9g, h).

### Lip

**Fig. 10:**
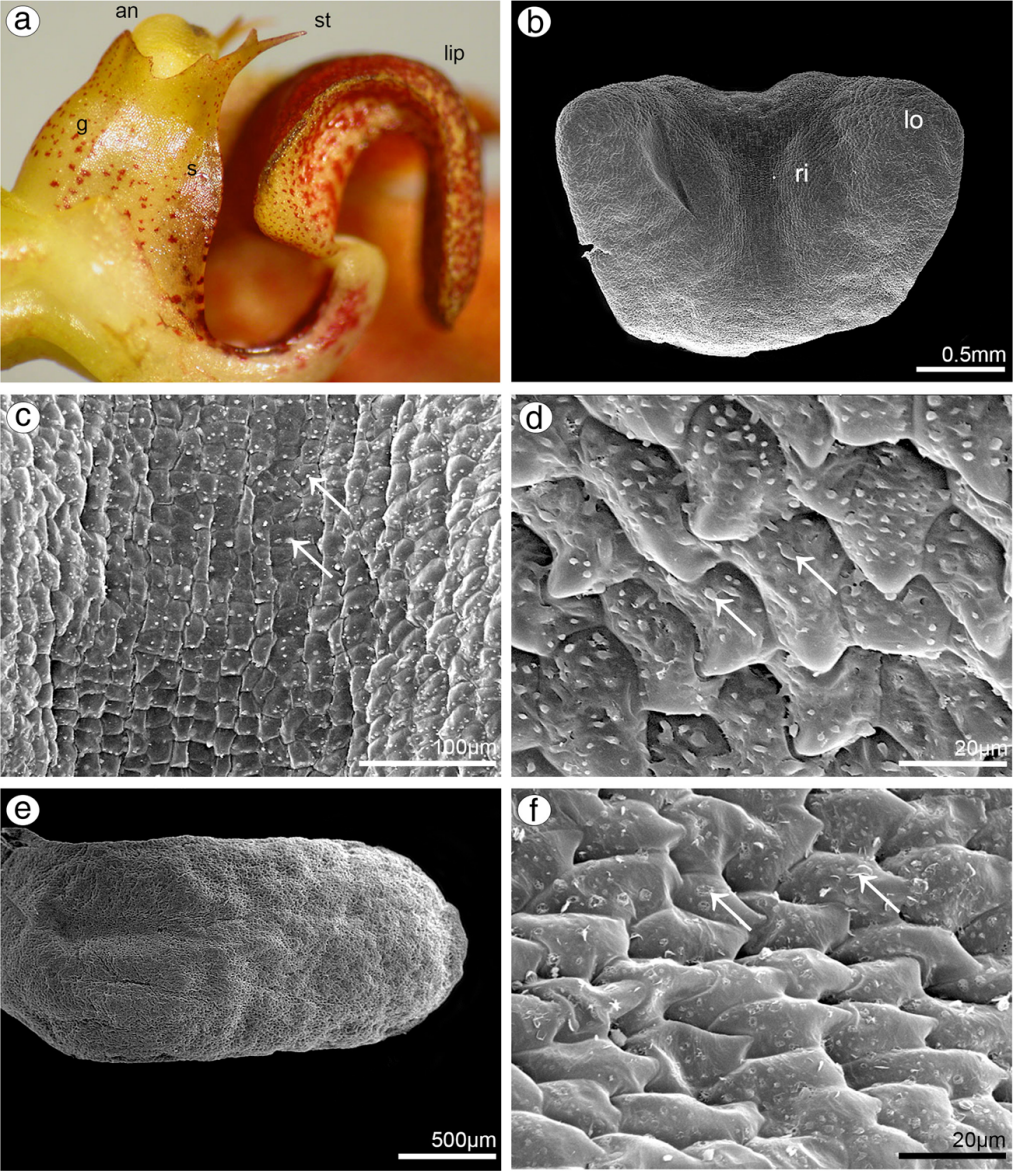
Macro- and micromorphological features of the lip of *B. weberi*. **a** Downwardly curved lip with gynostemium (*g*): anther (*an*), staminodium (*s*), and stelidia (*st*) (LM); **b** lip base with median longitudinal groove, surrounded by two weak ridges and raised lateral lobes (SEM); **c** groove cells with remnants of secreted material (*arrows*, SEM); **d** the central part of lip with cells placed imbricatelly towards the apex and secretory material (*arrows*, SEM); **e** lip apex (SEM); **f** detail of lip apex (**e**) imbricatelly positioned cells with remnants of secretion (*arrows*, SEM)

**Fig. 11:**
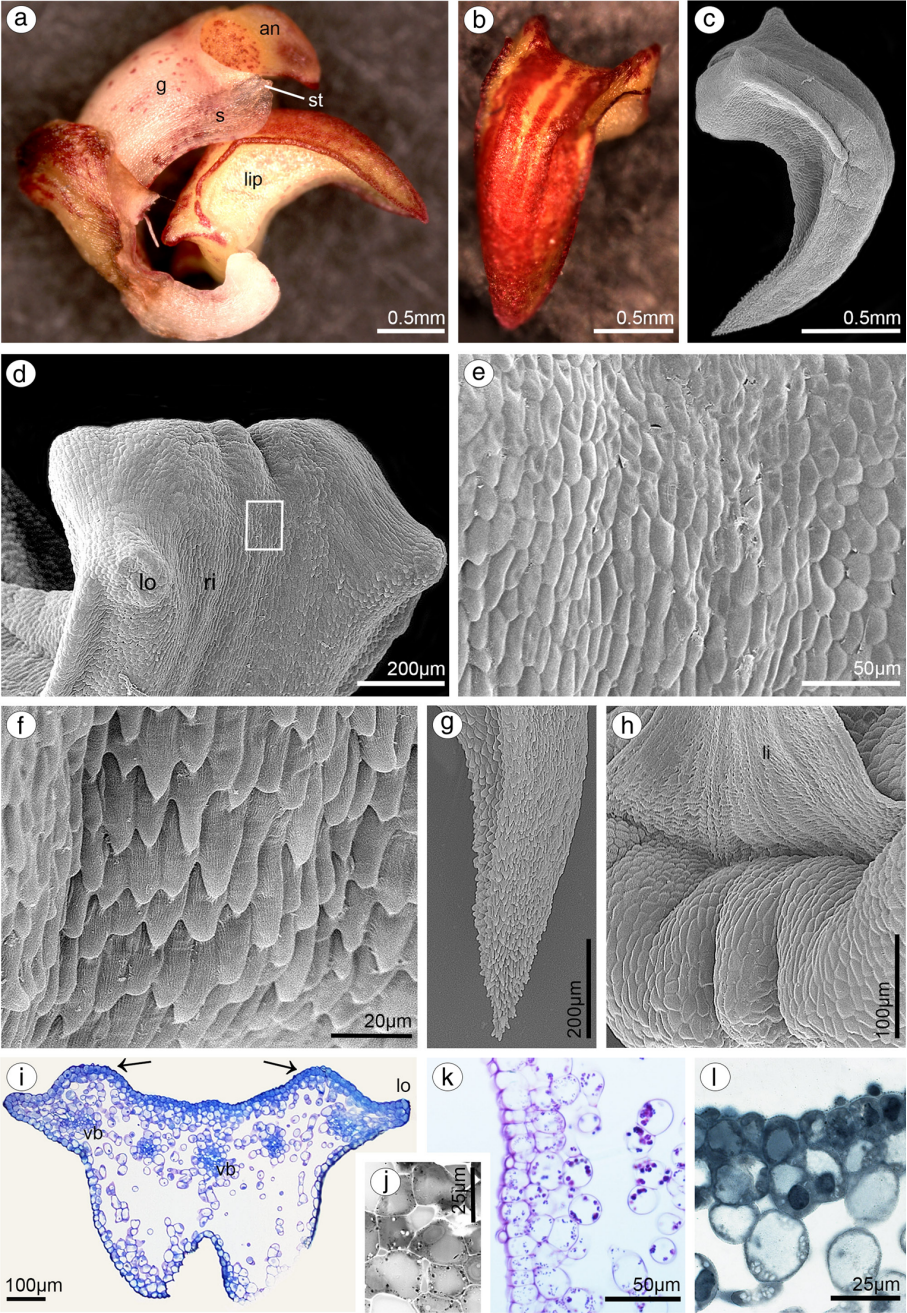
Macro-, micro- and anatomical features of the lip of *B. cumingii.* **a** Downwardly curved lip with gynostemium (*g*): anther (*an*), staminodium (*s*) and stelidia (*st*) (LM); **b** yellow with red stains lip apex (LM); **c** lip, lateral view (SEM); **d** lip base with median longitudinal groove, surrounded by two weak ridges and raised lateral lobes (SEM); **e** detail of lip base with median longitudinal groove (**d**) with few remnants of secretory material (SEM); **f** lip cells placed imbricatelly towards the apex (SEM); **g** lip apex with elongated papillae (SEM); **h** ligament (*li*) built by cells with striate ornamentation (SEM); **i** transverse section of lip base: median groove (*gr*), surrounded by two weak ridges (*arrows*) and lateral lobes (*lo*), few vascular bundles (*vb*) and large intercellular spaces (TBO); **j** few lipid droplets (SBB); **k** tiny starch grains (PAS); **l** the epidermal and subepidermal cells stained on proteins (ABB)

**Fig. 12:**
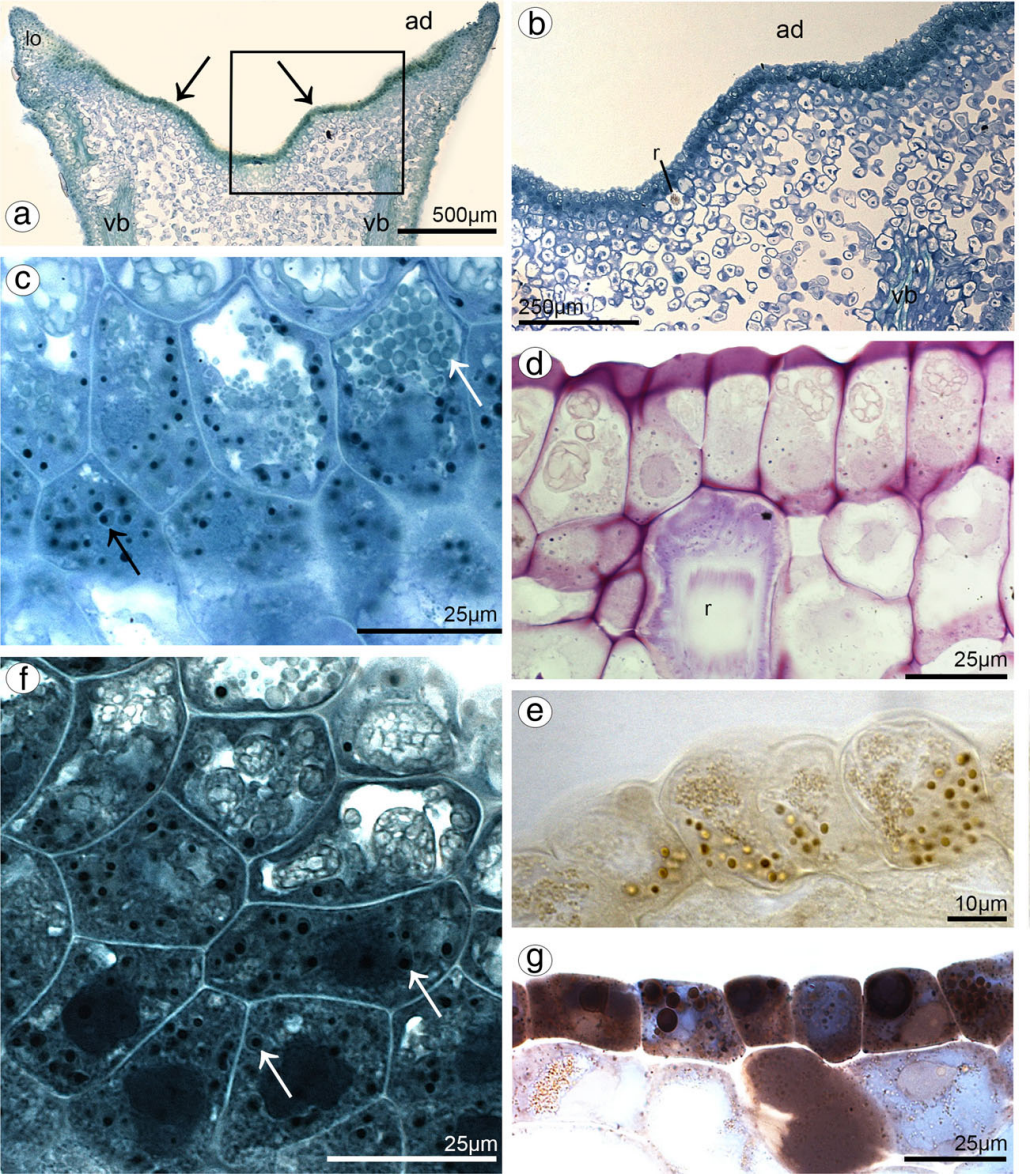
Histochemical features of the lip of *B. weberi.* **a** Transverse section: the median groove, surrounded by two weak ridges (*arrows*) and raised lateral lobes (lo), vascular bundles (vb), the intercellular spaces (TBO) (*ad* adaxial (inner) surface); **b** detail of transverse section (**a**), *r* idioblast with raphides in subepidermis (TBO); **c** subepidermal cells with two types of globules (*arrows*, TBO); **d** few, barely noticeable, starch grains (PAS), *r* raphides; **e** some globules stained on dihydroxyphenols (FeCl_3_) and some on proteins (ABB) (**f**); **g** epidermal cells strongly stained on lipids (SBB)

**Fig. 13:**
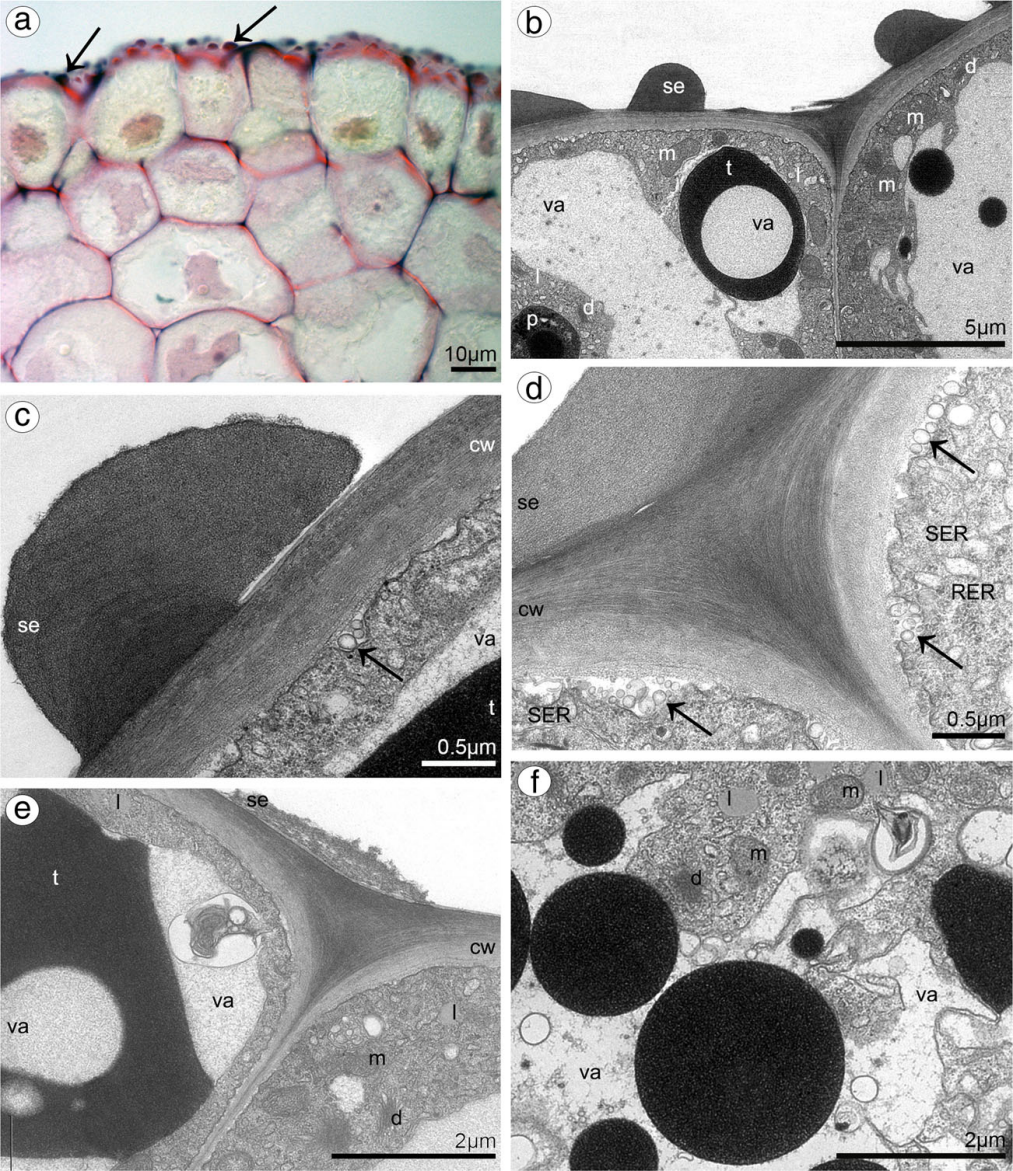
Features of the lip groove of *B. weberi.* **a** Transverse section of the lip with secretory material stained on pectic acids/mucilage (*arrows*) (LM, ruthenium red); the ultrastructural studies (TEM) revealed: secretory material (*se*) on surface (*cw* cell wall) (**b–e**); numerous mitochondria (*m*) (**b, d–f**), plastid (*p*) with electron-dense body (**b**); lipid bodies (*l*) (**a**, **e**, **f**), abundant smooth (SER) and rough endoplasmic reticulum (RER) (**d**), fully developed dictyosomes (*d*) (**a, d, f**), vesicles building into plasmalemma (*arrows*), plasmalemma with irregular outline (**c–e**), tannin-like materials (*t*) in vacuoles (*va*) (**b**, **c, e**); many osmiophilic globules in vacuoles (**b**, **f**)

**Fig. 14:**
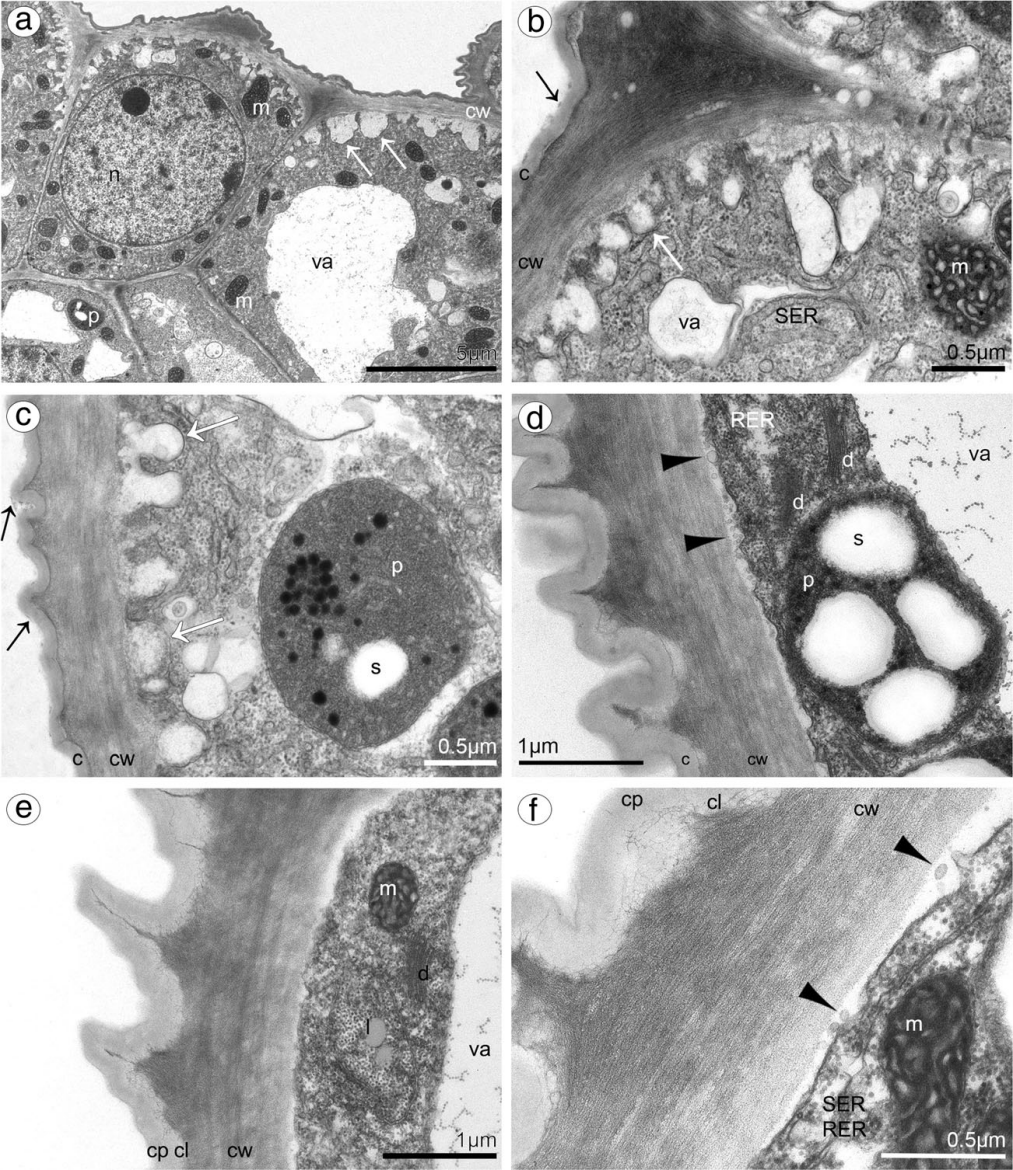
Ultrastructural features of the lip groove of *B. cumingii* (TEM): dense cytoplasm with large nucleus (*n*) and large numbers of mitochondria (*m*) (**a**); the ingrowths (*white arrows*) arising from the inner surface of the outer walls (**a–c**); abundant smooth (SER) and rough (RER) endoplasmic reticulum (**b, d, f**); few residues on cuticle (*black arrows*) (**c**); amyloplasts (*p*) with starch grains (*s*), plastoglobuli and irregular profiles (**c**, **d**); fully developed dictyosomes (*d*) (**d**, **e**); lipid bodies (**e**); few, occasionally noted, vesicles building into plasmalemma (*arrowheads*), plasmalemma with irregular outline (**d, f**); microchannels visible in reticulate cuticle layer (*c* cuticle, *cl* cuticle layer, *cp* cuticle proper) (**f**) (*cw* cell wall, *va* vacuole)

In both species, lip was thick and fleshy (Figs. 10a, 11a), arcuately down curved, yellow with red stains. Typically for bulbophyllums, the median longitudinal groove was present on the inner surface, additionally surrounded by two weak ridges and raised lateral lobes (Figs. 10b, 11b–d). The epidermal cells were placed imbricately towards the apex (Figs. 10d–f, 11f), forming the elongated papillae in the lip apex in *B. cumingii* (Fig. 11g). Secretion on the whole adaxial surface (on the groove and lobes) was detected in *B. weberi—*more in the groove (Fig. 10c, d, f), few in *B. cumingii* (Fig. 11d, e). The transverse sections displayed the anatomical lip features—the tissue consisted of one layer of epidermis, few subepidermal layers and ground parenchyma built by irregularly shaped cells, with intracellular spaces and vascular bundles (Figs. 11i, 12a). The histochemical staining showed that the epidermal and subepidermal layers of cells from the groove and lobes were stained more intensively in control staining—TBO (Figs. 10i, 11a–c) and for proteins in ABB staining (globules only in *B. weberi*—Fig. 12f, not in *B. cumingii* Fig. 11l). Treatment with SBB revealed that in *B. weberi*, the epidermal cells were strongly stained on lipids (Fig. 12g), whereas in *B.* cumingii, only few lipid droplets have been observed (Fig. 11j). The tiny starch grains were indicated in *B. cumingii* (Fig. 11k), while few, barely noticeable, in *B. weberi* (Fig. 12d). The two types of globules (Fig. 12c) appeared in TBO staining in epidermis and subepidermis of *B. weberi*. They were noticed in epidermis from two weak ridges towards the margins, and not found in the groove. The globules (with darker centre and lighter edge) were stained on proteins (Fig. 12f). Comparing with TEM results (Fig. 13b and with petal—Fig. 9g), in our view, the electron-dense body in plastids was the darker part. The second type (Fig. 12c—TBO, *white arrow*) were those ones which stained positively on dihydroxyphenols (Fig. 12e). The idioblasts with raphides, as in other tepals, were localized in subepidermis (Fig. 12b, d). The secretory material, detected in SEM (Fig. 10c–f), was stained on pectic acids/mucilage (Fig. 13a). Ultrastructurally, the secretory material was seen on lip groove in *B. weberi* (Fig. 13b–e); sometimes, few residues on cuticle were seen in *B. cumingii* (Fig. 14c). In the latter species, the ingrowths had been developed from the inner surface of the tangential walls as small protuberances (Fig. 14a–c). In both species, typical features of secretory tissue were detected as follows: dense cytoplasm with large nucleus (mainly centrally located Figs. 12f, 14a), numerous mitochondria, abundant smooth and rough endoplasmic reticulum, free ribosomes, fully developed dictyosomes and lipid bodies (Figs. 13b–f, 14a–f). The plastids in *B. weberi* (chromoplasts) had plastoglobuli, electron-dense body and irregular profiles (Fig. 13b). Whereas in *B. cumingii*, the amyloplasts were filled with starch grains (Fig. 14d). The starch was utilized and plastids with plastoglobuli and irregular profiles were detected (Fig. 14c). The noteworthy feature was the presence of vesicles building into plasmalemma in *B. weberi* (Fig. 13c, d), occasionally noted in *B. cumingii* (Fig. 14d, f). The vacuoles in the former species contained osmiophilic tannin-like material in vacuoles (Fig. 13b, e) and globular, osmiophilic bodies of various sizes (Fig. 13b, f). The microchannels were slightly visible in reticulate cuticle layer of *B. cumingii* (Fig. 14f).

### Gynostemium

In both species, the ligament (elastic structure joining lip base with column foot of gynostemium) (Fig. 11h) and column foots were composed of cells with striate ornamentation. The staminodes in *B. weberi* were erect, triangular, with elongated stelidia. Their margins had few, triangular teeth (Fig. 10a). In *B. cumingii*, the staminodes were erect, triangular, with entire margins, with reduced stelidia, obtuse to subacute at apex (Fig. 11a). The transverse sections of stelidia displayed no secretory function.

## Discussion

Flowers of *B. weberi* and *B. cumingii* are characterized by fly-pollinated features, i.e. colour, motile hair(s) at the tepals, motile and hinged lip. The overview of anatomical results upon both species revealed the secretory activity in the appendages and apices of dorsal sepals in both species (putative osmophores), petals in *B. weberi* (putative osmophores) and adaxial surface of lips in both species (it is not clear if they are superficial nectaries or other glands providing nutrients for pollinators). The petals of *B. cumingii* are rather inactive in secretion process. The lipid layer on the apices of lateral sepals of *B. cumingii* may protect the tissue.

The osmophore cells look like conical-papillate cells, described on the whole epidermis of petals in more than 200 species (Kay et al. 1981). In orchids, they can be localized in swollen apices of petals and/or sepals (van der Cingel 2001), on papillate labellar lobes in some Neotropical bulbophyllums (Teixeira et al. 2004) or could not be distinguishable from other floral parts (Stern et al. 1987; Vogel 1990). The surfaces of dorsal sepals in both species and petals in *B. weberi* were papillate and could function as osmophores. The term ‘unguentarius’—the floral organ bearing osmophores could be adopted here, as described in *Utricularia dunlopii* (Płachno et al. 2016). The fragrance constituents are probably synthesized in plastoglobuli. The plastids with plastoglobuli (also called osmiophilic bodies or plastidial lipids) occur in osmophores and nectaries (Figueiredo and Pais 1992; Stpiczyńska 1997, 2001). Then, the secretory products are transported outside the cell through the system of associated membranes: the intraplastidal ones, than plastid envelope, profiles of endoplasmic reticulum (or independently as lipophilic or osmiophilic droplets in cytoplasm) and finally—plasmalemma (Pridgeon and Stern 1985; Stern et al. 1987; Pais and Figueiredo 1994; Stpiczyńska 1997; Kowalkowska et al. 2012). In other orchids, the exudation is released through pores/cracks in *Restrepia* and *Restrepiella* (Pridgeon and Stern 1983), stomata in *Acianthera* (Melo et al. 2010) or through the cuticle reticulation (microchannels), which were slightly noticed in petal of *Bulbophyllum weberi*, previously described in osmophores of *Passiflora suberosa* L. (García et al. 2007), *Anacamptis pyramidalis* f. *fumeauxiana* (Kowalkowska et al. 2012), *Bulbophyllum wendlandianum* (Kowalkowska et al. 2015) and African *Bulbophyllum* species (Stpiczyńska et al. 2015). Micromorphological and ultrastructural studies revealed meagre amount of secretory material on petal surfaces of *B. weberi* and *B. cumingii*. Such feature was also detected in osmophores of *Stanhopea* (Stern et al. 1987; Vogel 1990), *Anacamptis* (Kowalkowska et al. 2012) and *Bulbophyllum* (Kowalkowska et al. 2015). The meagre accumulation on the surface is associated with the fact that fragrances are generally produced and released periodically (Stern et al. 1987; Vogel 1990), because of cytotoxicity of exudates.

On the surface of dorsal sepal of *B. weberi*, the secretory material covered small abaxial sunken unicellular trichomes situated in depressions in pairs (Fig. 2b) and stomata (Fig. 2f). Similar trichomes (but multicellular), accompanied by stomata, have been recorded on abaxial surface of dorsal sepals in Neotropical bulbophyllums section *Didactyle* (Nunes et al. 2014). In species from section *Didactyle*, on the basis of positive results for osmophoric activity (in vivo test) and slightly on phenolic compounds and pectic acids, they were interpreted as putative osmophores. The floral sunken trichomes have also been noted on tepals and labellum in *Maxillaria dichroma* Rolfe, and described as resin-secreting trichomes (Stpiczyńska and Davies 2009). Our histochemical results did not explain the function of such trichomes.

The chemical composition of fragrance may be mixtures of many components (Vogel 1990). Lipids, noted in other orchids, were considered the equivalents of fragrance production (Swanson et al. 1980; Pridgeon and Stern 1983; Curry et al. 1988) and were noted in both examined species in dorsal sepals and petals in *Bulbophyllum weberi*, yet not in *B. cumingii* (compare Figs. 4c, h; 5b; 9c). The osmiophilic irregular materials (Fig. 9f) were noticed in vacuoles and are probably tannin-like materials (Fig. 9e). Whereas globular, osmiophilic globules in vacuoles and sometimes in cytoplasm (Fig. 9f, g) could be formed from plastoglobuli (as they were placed close to plastids, Fig. 9g, *arrowheads*) and this suggests that they have lipoid character. They could also be tannin-like materials (globules from the test for dihydroxyphenols, Fig. 9e), the proteinaceous globules (the second type, Fig. 9d) or lipoid ones (Fig. 9c). Similar electron-dense globules also occurred in lip of *B. falcatum* (Lindl.) Rchb.f. and *B. maximum* Kraenzl. (Stpiczyńska et al. 2015). The first type of globules stained for proteins (darker central part and lighter edge) are, in our opinion, plastids with electron-dense body (Fig. 9d, g). The tiny starch grains were found in analysed species in PAS reaction. The starch grains, present in amyloplasts during the pre-secretory stage and hydrolysed at the anthesis stage (Stern et al. 1987; Curry et al. 1991; Melo et al. 2010; Pansarin et al. 2009; Antoń et al. 2012), are utilized as a source of energy in fragrance production (Vogel 1990). In summary, in the cells of dorsal sepals and petals of *B. weberi* proteins, dihydroxyphenols, lipids and starch grains were detected, in lateral sepal—lipids, whereas in dorsal sepal of *B. cumingii*—only lipids and starch grains, in lateral sepal—proteins and dihydroxyphenols, in petals—proteins and starch grains.

The lips in both examined *Bulbophyllum* species look similar on macro- and micromorphological levels: arcuately down curved, with the median longitudinal groove, surrounded by two weak ridges and raised lateral lobes. Nevertheless, histochemically and ultrastructurally they differ, but in both species, possibly function as putative nectaries. Secretions in *Bulbophyllum* are located superficially on lip grooves (described as nectaries) (van der Pijl and Dodson 1966; Vogel 1990; Borba and Semir 1998; Kowalkowska 2009, 2015). The secretory tissue in *Bulbophyllum* species (defined as ‘nectary’) generally comprises a secretory epidermal cell layer and few subepidermal layers (i.e., Teixeira et al. 2004; Nunes et al. 2014; Kowalkowska et al. 2015). The dense cytoplasm contains large amount of organelles, which evidence the highly metabolic function, present in secretory tissue. The numerous mitochondria, related to high metabolic cell activity, are found in nectariferous or osmophoric tissues (Pridgeon and Stern 1983; Stpiczyńska et al. 2005). The profuse ER and fully developed dictyosomes are involved in nectar secretion (Figueiredo and Pais 1992; Stpiczyńska et al. 2005). In orchid nectaries cells, osmiophilic substances (presumably lipid bodies) are often present (Stpiczyńska 1997; Stpiczyńska et al. 2004; Pais and Figueiredo 1994). The lipid bodies occurred in epidermal cells of lip in *B. rothschildianum* (Teixeira et al. 2004). In *B. weberi*, the lipid bodies were placed close to mitochondria, which can be associated with secretory process. The osmiophilic irregular materials (Fig. 13e) were noticed in vacuoles and these are probably tannin-like materials (Fig. 12e). The globular, osmiophilic globules in vacuoles and sometimes in cytoplasm (Fig. 13b, f) could be also tannin-like globules, detected in FeCl_3_ test (Fig. 12e). We did not interpret them as lipid bodies, as their character appears differently in cytoplasm (Fig. 12f), or protein bodies, as the globules stained for proteins (darker central part and lighter edge, Fig. 12f) are, in our view, plastids with electron-dense body (Fig. 13b).

The lip groove of *B. cumingii* differs from that of *B. weberi*. In the second species, the remnants of secretory material on surface indicated the positive result after treatment on pectic acids/mucilage. The mucilage was also detected in species from section *Racemosae* (Davies and Stpiczyńska 2014) and in cell walls of *B. falcatum* (Lindl.) Rchb.f. and on outer cell walls of *B. schinzianum* Kraenzl. (Stpiczyńska et al. 2015). The other difference between both examined species is presence of the ingrowths formed from the inner cell walls. Such ingrowths were also found in petals of *B. weberi*. For the first time, we described the cell wall ingrowths in *Bulbophyllum* species. The cell wall ingrowths are ubiquitous feature of plants, algae and fungi, found in various organs (Offler et al. 2003), also in nectaries (i.e. Stpiczyńska et al. 2012) and osmophores (i.e. Płachno et al. 2016). The cell wall protuberances may function as transfer cells—highly specialized cells, where an intensive transport of solutes through the plasmalemma is high (Gunning and Pate 1974). In nectaries of *Fritillaria meleagris* L. (Liliaceae), they appeared at the beginning of anthesis stage as small proturberances of the nectariferous epidermis and parenchyma formed from tangential walls of petals, and at full anthesis developed into labyrinthine protuberances. Such wall ingrowths, with the presence of microchannels or pores in cuticle, improve the nectar resorption. Resorbed nectar can be transported along the symplast and/or apoplast, and then is translocated across vascular tissue to the reservoir of assimilates (Stpiczyńska et al. 2012). The cell wall ingrowths were found in septal nectaries of *Tillandsia* (Fiordi and Palandri 1982), *Gasteria, Aloe* (Schnepf and Pross 1976) and *Strelitzia* (Kronestedt and Robards, 1987). In examined species, the cell wall ingrowths were prominent on the tangential walls of epidermal cells, but not in parenchyma cells—as recorded in *Fritillaria* (Stpiczyńska et al. 2012). Then, the nectar could pass across the cell wall through a permeable cuticle, modified stomata or pores/cracks in the cuticle (Nepi 2007). In the lip of *Bulbophyllum cumingii* and the petal of *B. weberi* the reticulate cuticle with microchannels and cell wall ingrowths may facilitate the secretion and resorption of secretory material, as it was speculated for *Fritillaria* (Stpiczyńska et al. 2012). However, the epidermal cells of *Bulbophyllum cumingii* had secretory activity (could be a superficial nectary), whereas cells in petal of *B. weberi* rather function as osmophore. The cell wall ingrowths described in *Epipactis atropurpurea*—species with hexose rich nectar, whereas in *Limodorum abortivum* were absent—species with sucrose dominant in nectar (Pais and Figueiredo 1994). According to such observations and comparing the results of both studied species, the nectar with sucrose dominant could be present in *Bulbophyllum weberi* and with hexose dominant in *B. cumingii*, but further studies are necessary to prove it. The dense cytoplasm with large amount of organelles in *B. cumingii* lip indicates the high metabolic activity, but only few remnants of secreted material were found on cuticle. The starch grains in plastids, which are hydrolysed during secretion (Stpiczyńska et al. 2004), were observed in both species. Moreover, the vesicles building into plasmalemma frequently distinguished in *B. weberi*, but few in *B. cumingii*, can designate transport of fragrance and/or nectar components in granulocrine secretion (Fahn, 1988) or other labellar secretory material (Stpiczyńska et al. 2015). In *B. cumingii*, the plastids did not have the electron-dense body and accordingly with it—the treatment with ABB did not show the presence of globules (darker inside, with lighter edge). The tannin-like material (dihydroxyphenols) found in cells of studied tepals offers protection against pathogens, herbivores and UV radiation. Tannins are described in cell suspension cultures and calluses from various gymnosperms (Constabel 1969; Chafe and Durzan 1973; Parham and Kaustinen 1977) or in petals (Ochir et al. 2010), forming in tannosomes (Brillouet et al. 2013). As flies are attracted by shining surfaces, calcium oxalate crystals could be found in lips with open nectaries (van der Cingel 1995), as described in *B. weberi*. Localized under the epidermal cells, they probably protect from herbivory (Prychid and Rudall 1999). Reassuming the histochemical treatments with several reagents of lip in *B. weberi* demonstrated that epidermal cells contain lipids, proteins, starch grains in cytoplasm, dihydroxyphenols in vacuoles and pectic acids/mucilage on surface. Whereas, in *B. cumingii*—few lipids, starch grains, no proteins, no dihydroxyphenols and no mucilage were noted. Ultrastructurally, we found in *B. weberi* secretory material on surface, while in *B. cumingii*—cell wall ingrowths and microchannels in cuticle layer.

As in other orchids (Stpiczyńska et al. 2004, 2005; Kowalkowska and Margońska 2009; Kowalkowska et al. 2015; Nunes et al. 2015), also in *B. weberi* and *B. cumingii*, lots of idioblasts with raphides of calcium oxalate were noticed in all tepals. The presence of idioblasts might be related to the elimination of calcium surplus from the cytosol (Paiva and Machado 2008). Furthermore, the large number of idioblasts in dorsal sepals and petals reflect the light and direct the attention of insects to the centre of flowers (van der Cingel 1995; Franceschi 2001). The floral attraction for pollinators could be increased by the presence of tepals striations, also described in species of *Bulbophyllum* section *Napelli* (Nunes et al. 2015) and in four African *Bulbophyllum* species (Stpiczyńska et al. 2015). The striations pattern may function as diffraction ratings: may create iridescence and act as cues for pollinators (Whitney et al. 2009).

In conclusion, the examined species, besides the macromorphological similarities, greatly vary on anatomical and ultrastructural level. More studied *Bulbophyllum* species could give the insights into this orchid group and likewise indicate features, which could be taxonomically important.
